# Hospitals and the Poets.—V. Dr. Erasmus Darwin, Scientist, Physician, and Poet

**Published:** 1916-03-04

**Authors:** 


### HOSPITALS AND THE POETS.*

### V. Dr. Erasmus Darwin, Scientist, Physician, and Poet.

In his own day (1731-1802) Dr. Erasmus Darwin, whose grandson wrote the "Origin of Species," was less popular as a scientist than as a physician and a poet. To-day his reputation as one of the founders of evolution is receiving its due—thanks largely to Samuel Butler's resuscitation of those passages in the *Zoonomia* which reveal Dr. Darwin as an evolutionist who holds that "the design which has designed organisms, has resided within, and been embodied in, the organisms themselves." On the other hand his poems seem to us like parodies of themselves, grotesque attempts to make poetry by explaining in verse the nature of electricity or the principle of the pump. It is easy now to laugh at Dr. Darwin's poems, and the literary critic to-day, with a superior air, can make merry over his attempt to "enlist Imagination under the banner of Science." But he did not fail in spite of the ludicrous lines in his "Loves of the Plants," for anyone who will turn from his verse to his prose will find that if he did not enlist imagination under the banner of science and make poetry out of the facts of botany, he did enlist science under the banner of the imagination and thereby raise it from an academic speculation into a philosophy of life. The result was that his prose had the virtues which his verses lacked, so that he was a poet in the sense that the body of his work was poetry.

To prove the foregoing, since the writer's object is to show that Dr. Darwin went nearer to achieving his aim than at first sight seems possible, the following quotations must suffice. In his poem the *Botanic Garden*, which was divided into two parts, "The Loves of the Plants" and "The Economy of Vegetation," he could write such lines as:

> Beard the bright cylinder with golden wire
> And circumfuse the gravitating fire; which is descriptive of electricity! Many more are really too ludicrous to quote. Their essential quality, however, is given with imperishable fun in the following lines, intended as a parody of "The Loves of the Plants," which were written by Frere and published in the *Anti-Jacobin* under the title of "The Loves of the Triangles."

> Fair Hydrostatics, simpering as they go,
> Lead the light Naiads on Fantastic toe;
> Th' obedient pulley strong Mechanics ply,
> And wanton Optics roll the melting eye.

That is the worst that can be said of Dr. Darwin's verses, and that he delighted in the parody is sufficient to assure the experienced reader that the verses were the weakness of a mind whose scope was not exhausted by them.

### Can Animals Enter into Contracts?

As an instance of Dr. Erasmus Darwin at his best let us reproduce from the *Zoonomia* this passage. Butler quotes it as indicating Darwin's readiness to take common words according to their common meanings and to connect things and qualities which resemble one another, if after inspection they are seen to do so, in spite of any theories presuming to stand in the way. Thus Dr. Darwin writes:

> An ingenious philosopher has lately denied that animals can enter into contracts, and thinks this an essential difference between them and the human creature: but does not daily observation convince us that they form contracts of friendship with each other and with mankind? When puppies and kittens play together, is there not a tacit contract that they will not hurt each other? And does not your favourite dog expect you should give him his daily food for his services and attention to you? And thus barters his love for your protection? In the same manner that all contracts are made among men that do not understand each other's arbitrary language.

### A Penetrating Insight.

If one quality of the poet's mind be to show us analogies between things in which we had not suspected them hitherto, and thereby to make us aware of the spirit which alone gives life to the letter of our words and definitions, then Dr. Darwin, like the able scientific thinker that he was, shared at least one quality with the poet. He insisted on the oneness of personality between parent and offspring, which last, he remarked, was merely "an elongation of the parent," and thereby he was led to regard instinct as "reason become habitual"; he was convinced of "the essential unity of animal and vegetable life," and argued that "the individuals of the vegetable world may be considered as inferior or less perfect animals." He believed that the voluntary power of vegetables, as shown, for instance, in the circular movement of vine-tendrils, was apparent in their "necessity to sleep." In considering the way in which "the anthers in many flowers, and stigmas in other flowers, are directed to find their paramours," he asks, "Is this curious kind of *storgé* produced by mechanic attraction or by the sensation of love? The latter opinion [Dr. Darwin concludes] is supported by the strongest analogy, because a reproduction of the species is the consequence." A few lines further on we find him saying: "Vegetable life seems to possess an organ of sense to distinguish the variations of heat, another to distinguish the variations of moisture, another of light, another of touch, and probably another analogous to our sense of smell. To these must be added the indubitable evidence of their passion of love, and I think we may truly conclude that they are furnished with a common sensorium for each bud, and that they must occasionally repeat those perceptions, either in their dreams or waking hours, and consequently possess ideas of so many of the properties of the external world and of their own existence." The ideas contained in the fine passage of which this is but an excerpt, have not only been endorsed by modern scientists, but

* The first article in this series appeared on page 329 of The Hospital for January 8, the second on page 369 for January 22, the third on page 409 for February 5, and the fourth on page 453 for February 19.

stand for us as proof that, though he could not clothe in forms of living poetry all that his inner eye perceived, the sight, the vision, was in him, and this allows us to read, as it were, the poetry between the lines of his prose.

These quotations, which may be studied in full in the chapters devoted to Dr. Darwin's work in Samuel Butler's *Evolution Old and New*, have proved so fascinating that the space which, according to the letter of these articles, ought to have been devoted to his life as a physician, has been encroached upon. It remains therefore to relate something of his life as a medical practitioner, (which has been told with much "unconscious humour" by Miss Seward), but much more briefly even than Butler was compelled to do.

### Cambridge, Edinburgh, London.

Born in 1731, Dr. Darwin was the son of a Nottinghamshire gentleman with a taste for literature and science, and was educated at St. John's College, Cambridge, where he graduated M.B. in 1755. In the previous year he had taken his B.A., and composed a poem on the death of Frederick, Prince of Wales, before departing for Edinburgh to begin the study of medicine. His term of study seems to have been short, for not only did he graduate in medicine the next year, as stated, but after attending Dr. Hunter's lectures in London, started practice as a physician in Nottingham. He was so unsuccessful that he promptly moved next year, 1756, to Lichfield, where he had the good luck to be called in to attend a young man of good position who had been given up as hopeless, we read, by the leading doctor of the place. By the employment of what one biographer has called "a reverse and entirely novel kind of treatment" the patient recovered, and young Dr. Darwin was soon making £1,000 a year! That he had found his feet may be judged from the fact of his marriage, which took place within a year of this piece of good fortune, and for the rest of his life Dr. Darwin's practice continued to prosper and his reputation steadily increased. A kind of Dr. Johnson in appearance, who held his own court, by the way, under the nose of his more famous rival in Lichfield, to whose dictatorship he refused to bow, Dr. Darwin was a great, in fact an enormous, eater. He was no glutton, abstemious to a degree in the use of wine, but he believed in a superabundant diet, and attached very great value to the nutritive power of sugar, consuming sweetmeats, cream, sugar, and fruit as he drove on his rounds in a great carriage, in which writing materials, books, and a hamper were always packed. He was an open-handed host, generous to the poor, and attended without fee many people, including the poorer cathedral clergy. But general practice did not exhaust Dr. Darwin's energies. Though poetry and science occupied partly the earlier, but mainly the latter, years of his life (the *Botanic Garden* appeared from 1789-1792), and the *Zoonomia* was published as late as 1794-1796, Dr. Darwin was always a keen botanist, and student not only of Nature but of art, especially mechanics, in which he tried his hand as an inventor. He constructed a strange carriage, of complicated and obscure design, if we may judge from the descriptions that have come down to us, and from this he had a fall in 1768, breaking the patella of his right knee and suffering a slight lameness ever afterwards. His attitude to inventions may be guessed and approved from his definition of a fool as "a man who never tried an experiment in his life."

### Dr. Darwin as a Tub-Thumper.

His wife having died in 1770, he married again in 1781, moving to Derby and then to Breadsall Rectory, where he died. Such poetry as he wrote in the middle of his life was composed while visiting his patients, and his care for those of them who were poor is shown by the dispensary which he founded at Lichfield in 1784. He once gave a remarkable address from a tub in the centre of Nottingham Market to a crowd of listeners on the value of open windows and fresh air, an address which was doubtless given point to by the dripping clothes in which the speaker stood, for he was inspired to this speech, without his usual stammer, after having swum across a river from a boat in which he and a few friends were taking a rowing expedition one summer day soon after his having settled at Lichfield The address, a model medical lecture to a lay audience which school medical officers and doctors in the public service would do well to study, is easily accessible in Butler's pages, where it forms part of Miss Seward's delicious portraiture.

### A Pioneer of Evolution.

His many interests, his humour, his kindliness, his restless curiosity made science to him no specialised department or isolated province of research, but part and parcel of philosophy, so we find him in 1797 producing "a plan for the conduct of female education in boarding schools," and in 1797 engaged on "a Philosophy of Agriculture," while after his death appeared the poem entitled *The Temple of Nature*, in which the origin of society was discussed. His great work is, of course, the *Zoonomia*, in which the teleological view of evolution, first put forward, in a disguise of transparent irony, by Buffon, to be developed afterwards by Lamarck and Butler (in contradistinction to Charles Darwin's theory of natural selection), is implicit, if not fully expressed or understood. But he stands for us to-day not only with Buffon and Lamarck as one of the three founders of the theory of evolution, but as an able, almost a great, man, in whom the detailed knowledge of the expert in botany and zoology only served to heighten the sense that Nature is "a temple," wherein men and animals and plants alike watch and pray by the twin processes of instinct and intelligence. These being differences of degree rather than of kind, by proving some power of adapting self and surroundings to each other enable us to state that "one and the same kind of living filament is and has been the cause of all organic life."

